# 模拟放疗引导穿刺染色定位切除外周肺部微小结节

**DOI:** 10.3779/j.issn.1009-3419.2016.09.03

**Published:** 2016-09-20

**Authors:** 锋 毛, 良 张, 恒乐 顾, 辉 张, 长兴 吕, 屠阳 申

**Affiliations:** 1 200030上海，上海交通大学附属胸科医院/上海市肺部肿瘤临床医学中心胸外科 Department of Lung Tumor Clinical Medical Center Surgery, Shanghai Chest Hospital of Shanghai Jiaotong University, Shanghai 200030, China; 2 132000 长春，吉林省肿瘤医院胸部肿瘤科 Department of Chest-Oncology, Jilin Province Tumor Hospital, Jilin 132000, China; 3 200030 上海，上海交通大学附属胸科医院放疗科 Department of Radiotherapy, Shanghai Chest Hospital of Shanghai Jiaotong University, Shanghai 200030, China

**Keywords:** 肺部周围型微小结节, 放疗计划系统, CT模拟定位, 亚甲蓝注射, 楔形切除, Small pulmonary nodule, Radiotherapy planning system, Puncture under CT location, Methylene injection, Wedge resection

## Abstract

**背景与目的:**

随着肺癌早期筛查的广泛开展及高分辨率计算机断层扫描（comouted tomography, CT）的普及，临床上微小结节型肺癌在可手术的肺癌中所占的比例大幅增加。如何在术中快速而准确地找到病灶是近年来胸外科医生经常面临的问题。为此我们开展此项研究，旨在探索利用放射治疗计划系统模拟引导穿刺染色定位外周肺部微小结节，并评估这一新方法的定位效率和临床应用价值。

**方法:**

2012年2月-2015年1月我们对97例患者共100枚直径1 cm内的肺部周围型微小病灶，术前利用放射治疗计划系统，对病灶进行CT模拟定位，患者麻醉后依据术前放疗计划标记的穿刺位点、角度和深度，向病灶注射亚甲蓝，继而手术，在胸腔镜下根据染色标记楔形切除病灶区域肺组织。剖视标本并快速病理检查。统计穿刺注射亚甲蓝的时间、染料注射完毕至剖胸寻视到染色斑的时间、色斑中心点与病灶边缘的距离、定位成功率和并发症率等数据。

**结果:**

100枚病灶共完成定位96枚，成功率为96%。麻醉后皮肤定位点穿刺注射亚甲蓝的时间为（4.85±1.25）min；染料注射结束至入胸寻找到染色斑的时间为（16.36±2.36）min；色斑中心点与病灶边缘的距离为（4.78±2.34）mm；所有患者无并发症。

**结论:**

放疗计划系统模拟定位引导穿刺亚甲蓝注射法对肺部周围型微小结节的定位成功率较高，无相关并发症。此方法可避免患者清醒状态下穿刺定位的恐惧和疼痛，并可明显减少患者的辐射危害。

## Abstract (en)

**Background and objective:**

With the extensively application of HRCT (high resolution CT) and the popularization of early lung cancer screening, the proportion of small nodullar lung cancer to be operated increases rapidly. Identifying the focus lesions quickly and accurately in operation has shown to be a challenge. We carried out this research trying to make use of and evaluate a new method that localizaes and extracts small peripheral pulmonary nodules by way of simulating radiaotherapy combining methylene blue staining.

**Methods:**

From February 2012 to January 2015, 97 patients with 100 peripheral pulmonary nodules ≤10 mm in size were simulated puncturing using a radiotherapy planning. When the anaesthesia came into use, methylene blue dye was injected to the virtually identified point corresponding to the surface point, according to the angle and depth previously computed by the radiotherapy planning. The video-assisted thoracoscopic surgery (VATS) wedge resections of the marked lesions were undertaken and the specimens were sent for frozen pathologic examination. The interval time from anesthesia-completing to puncture and injection, The interval time from methylene blue injection to identifying the stained area and the distances between the centre point of the stains and edge of coloured lesion were recorded.

**Results:**

Our preoperative localization procedure was successful in 96 of 100 (96%) nodules. The interval time from anesthesia-completing to puncture and injection of methylene blue were (4.85±1.25) min. The interval time from methylene blue injection to identifying the stained area was (16.36±2.36) min. The distances between the centre point of the stains and edge of coloured lesion were (4.78±2.51) mm. No complication was observed in all participants.

**Conclusion:**

The new method of locating peripheral pulmonary nodules by simulating simulating radiaotherapy combining methylene blue staining has a high success rate and no complication for localizing small peripheral pulmonary lesions, avoiding the fear and pain of the patients untaken puncture without anaesthesia reducing radial damage.

肺部微小病灶中有相当比例的早期肺癌^[1]^，据报道^[2]^单纯型磨玻璃样病变的恶性率高达75%以上。对肺部周围型微小病灶，原则上应先行病灶楔形切除，根据术中病理性质决定最终的切除范围，以避免良性病变的肺叶切除。但在实际操作中，如何在术中快速而准确地找到切除目标，特别是手指难以触及的微小病灶，是胸外科医生经常面临的挑战。迄今报道的多种术前及术中定位方法，利弊互现，均有一定的局限性。为此，我们创新了一种定位手段：模拟放疗引导穿刺染色法，对该方法的定位效率及其安全性进行了观察和评估。

## 1 资料与方法

### 1.1 病例资料

2012年2月-2015年1月，我们选择97例患者100例直径在10 mm内的肺部周围型微小病变进行了研究。纳入标准：①要求术前所有患者均已行胸部计算机断层扫描（computed tomography, CT）检查，测量病灶最大直径小于10 mm、位于肺野外带、判断术中肺表面不能看到病灶、能够行肺楔形切除手术；②术前病灶性质不明，无病理学诊断；③术前血常规、肝肾功能、电解质、血糖、凝血功能、心电图及肺功能等指标无明显异常，腹部B超（包括肝和肾上腺）、头颅磁共振成像（magnetic resonance imaging, MRI）及全身骨扫描排除远处转移。

排除标准：①病灶最大直径大于10 mm较易定位者；②病灶位于肺野中、内带，距离胸膜2.5 cm以上不宜行楔形切除手术者；③病灶紧贴脏层胸膜、胸膜凹陷明显，估计术中较易窥及病灶者；④术前根据胸部CT估计穿刺时会有肩胛骨或其它骨质结构遮挡，无法注射亚甲蓝者；⑤病灶离心脏、大血管、膈肌和神经组织较近，穿刺可能发生损伤，风险较大者。

患者均因体检CT扫描时发现肺部结节或阴影，就诊于上海市胸科医院/上海市肺部肿瘤临床医学中心。其中男性44例，女性53例，年龄26岁-81岁，平均年龄（51±14）岁。病灶直径4 mm-10 mm，平均（8.23±2.67）mm。病灶与胸膜的平均距离为（11.43±5.03）mm。其中，右肺上叶病灶25枚，右肺中叶15枚，右肺下叶20枚；左肺上叶19枚，左肺下叶21枚。患者于术前一天进行CT扫描采集影像，利用放疗计划测算模拟CT穿刺定位的进针点、角度及深度。

### 1.2 定位程序

#### 1.2.1 图像采集

研究者于术前一天陪护患者至我院放射科CT室，在德国西门子公司生产的Siemens SOMATOM Sensation 16型CT扫描机下行胸部薄层扫描（层厚2 mm）。患者体位原则上要求使其感到舒适、易于保持、方便重复。根据扫描前已有的CT资料，对可能的穿刺方向做出预判断，并据此调整好患者的体位。体位分为两种：①仰卧位：患者仰卧于检查床，双手掌心向上，右手重叠于左手之下合拢，抱头于枕部（图 1A）。此体位适合于拟从胸前或腋区穿刺注射亚甲蓝者，且在患者麻醉后最易被动重复；②俯卧位：患者俯卧于检查床，两臂自然下垂，手指并拢贴于腿侧（图 1B）。此体位适合于拟从背部穿刺注射亚甲蓝者。患者摆好体位后，打开CT机定位激光，研究者用水笔在患者胸部标记定位激光线，激光线呈三个十字交叉，交叉点位于同一平面，以此来确定原始定位平面，并在三个交叉点上贴敷金属标记点以确定。开始CT扫描时嘱患者深吸气后屏气，以减少呼吸动度对病灶扫描图像的影响。

**1:**
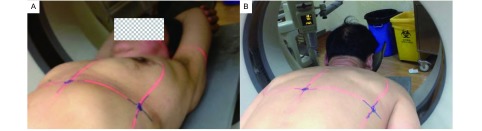
患者行胸部CT扫描姿势。A：仰卧位；B：俯卧位。 Position of patients underwent CT scan. A: Supine position; B: Prone position. CT: computed tomography.

#### 1.2.2 图像传输及模拟穿刺

将放射科CT室采集的患者扫描图像传输到我院放射治疗科，以德国IBA公司生产的Philips-Pinnacle 3放射治疗计划系统制定穿刺计划。该系统原本用于胸部肿瘤的放射治疗计划的制定，我们借用该系统的功能模拟穿刺定位的诸元素，可精确测定病灶所在胸部横断层面与CT扫描时原始定位层面的距离、放射治疗射线投射向病灶中心的方向和深度、射线从皮肤到病灶中心的距离及病灶边缘与胸膜的距离。我们设想以射线模拟穿刺针，那么射线在皮肤的投射点即为穿刺点，射线方向即为进针角度，皮肤投射点至病灶中心的距离即为进针深度。在治疗计划系统的图像模拟设计中，可提供不同皮肤入射点的相应入射角度和深度数据。因而我们可以选择合适的皮肤进针点，以避开肋骨和血管神经组织。进针角度宜选择水平或垂直位，以避免在实际穿刺时进针角度控制的困难，减少定位误差（图 2）。记录模拟穿刺的数据。

**2:**
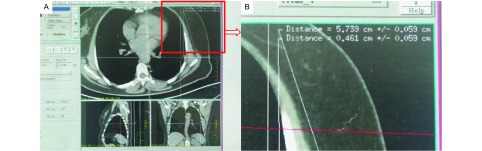
放射治疗计划系统屏幕截图。A：设定为水平穿刺测得的进针深度；B：局部放大。 Screenshot of radiotherapy planning system. A: Depth of needle using horizontal angle; B: Amplification of the area in the red squrel.

#### 1.2.3 穿刺点标定

研究者陪护患者至我院放射治疗科，在美国瓦里安公司生产的Ximatronc-series模拟定位机上重复CT扫描时的体位，使模拟机上的控制激光线与患者图像采集时标记的三条十字交叉线重合，以保证此刻体位与图像采集时一致（图 3）。在放射治疗计划系统图像模拟穿刺数据的引导下，通过计算机控制机架和治疗床的运动，定位激光照射患者胸部皮肤的光点即为穿刺进针点，以记号笔标记米字（图 4）。

**3:**
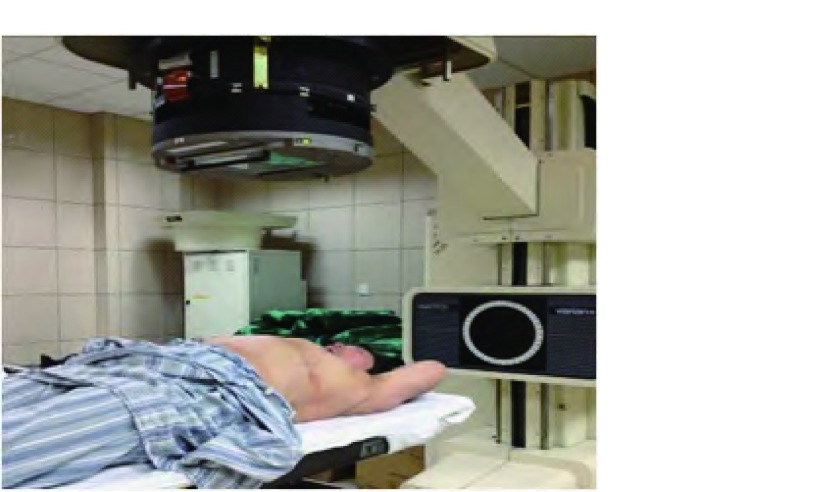
模拟机上复制CT扫描时的体位 Re-relization the position of CT scan on analog machine in the red squrel.

**4:**
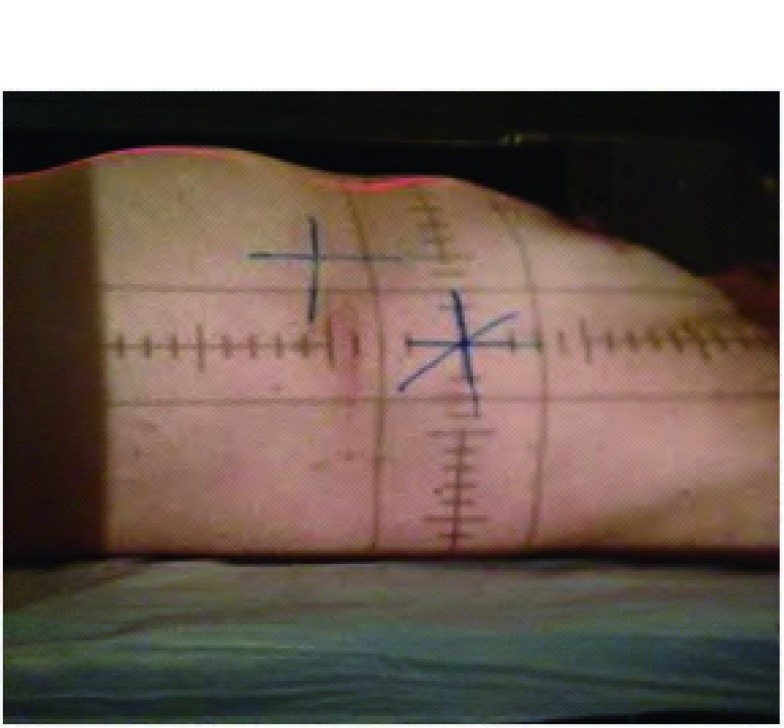
皮肤标记穿刺点 Puncture point marked on the skin

#### 1.2.4 注射亚甲蓝标记

患者麻醉后，人工控制患者体位，保持与术前CT图像采集时一致。消毒记号笔标记的穿刺点皮肤，根据治疗计划系统提供的进针角度和深度数据，在皮肤标记点进针，穿刺至病灶中心深度时注射亚甲蓝0.3 mL，逐步退针距脏层胸膜10 mm时再注射亚甲蓝0.2 mL。注射期间以呼吸机控制患者呼吸在深吸气时屏气状态，每次注射前回抽注射器无回血以避免注入血管（图 5A、图 5B）。穿刺针规格：20 G×150 mm，浙江日医有限公司生产。亚甲蓝20 mg/支，江苏泰州济川制药厂生产。注射亚甲蓝1 mL注射器规格为0.45 mm×16 mm，由上海康德莱企业发展集团股份公司生产。

**5:**
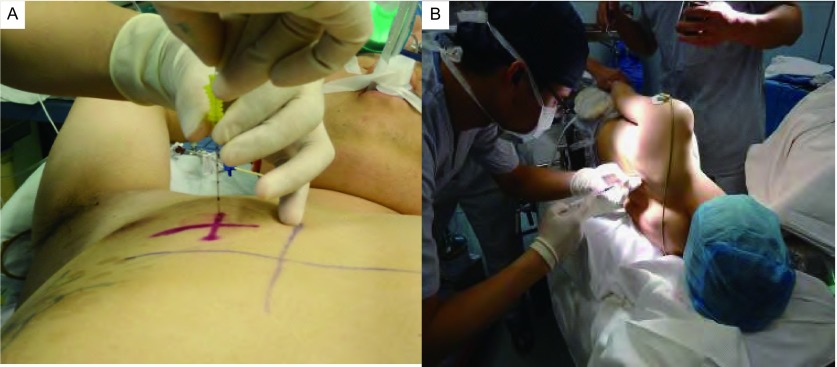
麻醉后放置模拟体位向病灶注射亚甲蓝。A：仰卧位垂直注射；B：侧卧位水平注射。 Puncture and methyblue staining after anesthesia-completing. A: Supine position; B: Lateral position.

### 1.3 手术方法

注射亚甲蓝结束，即刻摆好患者手术体位，消毒剖胸，使用德国Karl Storz公司生产的HD endoscopy胸腔镜探查肺表面亚甲蓝染色位置，窥及染色斑者，以手指在染色斑附近触摸查找病灶并楔形切除，切除范围以病灶为中心，包括染色斑在内，切缘距病灶边缘超过3 cm。取出标本，触摸病灶，以手术刀将病灶对称剖开，标本旁置一次性无菌5 mL注射器作为长度标尺，注射器规格0.7 mm×32 mm，由上海康德莱企业发展集团股份公司生产。使用日本SONY数字产品有限公司生产的DSC-W730型数码相机拍照记录后送检标本。如术中病理为浸润性肺癌，继续行病灶所在肺叶切除及系统性淋巴结清扫术，良性病变病例则结束手术。部分病例病灶的CT影像、胸腔镜下所见及楔形切除标本剖检照片见图 6。

**6:**
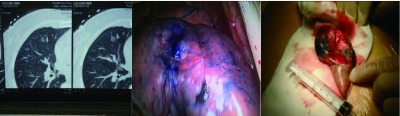
右肺中叶内段病灶CT影像、胸腔镜下所见及楔形切除标本剖检 CT image of the focus on the right middle lobe lung, screenshot of VATS, visual examine of the specimen

### 1.4 统计学方法

使用SPSS 19.0软件进行分析。统计分析研究测定的所有数据，包括定位时间，分为术前（患者从CT扫描至皮肤标定结束的时间）和术中（注射亚甲蓝结束至胸腔镜入胸的间隔时间）、病灶平均直径、病灶边缘距脏层胸膜的最近距离、定位边距（染色斑边缘距病灶边缘的最近距离）等数据以Mean±SD表示。定位成功率、并发症发生率以百分率表示。

## 2 结果

97例患者的100例结节定位成功96例，成功率96%。无定位操作相关并发症。麻醉后皮肤定位点穿刺注射亚甲蓝的时间为（4.85±1.25）min；染料注射结束至入胸寻找到染色斑的时间为（16.36±2.36）min；色斑中心点与病灶边缘的距离为（4.78±2.34）mm，详见表 1。所有患者术后恢复顺利，无并发症，如期出院。

**1:** 病例相关定位数据及其病理类型（仅为定位成功者） Data of location and pathological types (success cases only)

Lesion site (*n*)	Time (min)		Positioning margin (mm)	Pathological types
	Pre-operation	Inter-operation		
Right lung				
S2 (*n*=10)	5.30±1.25	16.30±3.20	4.00±1.89	IA (*n*=5), AAH (*n*=2), AIS (*n*=2), MIA (*n*=1)
S3 (*n*=15)	4.60±1.30	16.00±1.41	4.00±1.75	IA (*n*=9), AAH (*n*=2), AIS (*n*=2), MIA (*n*=2)
S4 (*n*=9)	5.22±1.39	16.78±1.86	4.30±0.91	IA (*n*=3), AAH (*n*=2), Meta-tumor (*n*=1), LN (*n*=3)
S5 (*n*=6)	5.17±1.72	15.50±2.07	3.10±0.71	IA(*n*=3), AAH (*n*=1), MIA (*n*=1), LN (*n*=1)
S6 (*n*=4)	5.00±1.41	18.33±1.25	10.00±2.65	IA (*n*=2), MIA (*n*=2)
S7-10 (*n*=14)	4.79±1.89	16.64±2.59	6.36±3.04	IA (*n*=7), Sarcoidosis (*n*=1), LN (*n*=1), Fop (*n*=1), AIS (*n*=2), MIA (*n*=2)
Left lung				
S2 (*n*=3)	5.00±0.00	15.67±2.08	6.67±1.15	IA (*n*=1), Hamartoma (*n*=1), AIS (*n*=1)
S3 (*n*=12)	4.58±1.62	16.67±2.64	3.67±1.31	IA (*n*=8), AAH (*n*=1), AIS (*n*=2), Granuloma (*n*=1)
S4+5 (*n*=4)	4.25±1.26	15.75±2.50	3.00±0.00	IA (*n*=1), AAH (*n*=1), AIS (*n*=1), MIA (*n*=1)
S6 (*n*=2)	5.67±0.58	14.67±3.79	1.50	AIS (*n*=1), MIA (*n*=1)
S7-10 (*n*=17)	4.72±1.07	16.44±2.64	5.88±2.85	IA (*n*=7), LN(*n*=1), Granuloma (*n*=2), MIA (*n*=4)，AIS (*n*=2), AAH (*n*=1)
Total (*n*=96)	4.85±1.25	16.36±2.36	4.78±2.34	
Pre-operation locating time: the interval time from anesthesia-completing to puncture and injection of methylene blue. Inter-operation locating time: the interval time from methylene blue injection to identifying the stained area. Position margin: The distances between the centre point of the stains and edge of coloured lesion.				

## 3 讨论

目前，对肺部周围型微小病灶有多种定位方法，一般可分为术前或术中定位两大类。术前定位方法包括：①金属钩穿刺定位法1992年Plunkett等^[3]^开始进行在CT下用金属钩对肺部周围型结节定位的研究和实践。此定位技术准确率较高，但当病灶距脏层胸膜较近时肺萎陷后金属钩较容易发生移位导致定位失败，其他的并发症主要包括气胸、胸痛、咯血及胸膜反应等^[4, 5]^，个别病例曾出现断针残留^[6, 7]^。另外，此方法对患者及操作人员的辐射较多，在一定程度上也会增加患者的心理负担。同时，过程中要求CT室和手术室密切配合，确保定位后立即进行手术，这种运作的方式在繁忙的医院也有一定的困难。②亚甲蓝染色定位法1994年，Lenglinger等^[8]^尝试术前在CT引导下使用穿刺针注射亚甲蓝对病灶进行染色定位，获得了比较满意的效果。此方法排除了与金属钩相关的并发症，但须注意选择合适的患者。年龄较大或长期吸烟的患者，其肺泡内碳末沉积，肺表面颜色变深，脏层胸膜颜色与亚甲蓝的着色相仿，有时会导致亚甲蓝识别困难。同时也需要保证手术的不间断性，如果注射后不能随即进行手术，则亚甲蓝在肺表面迅速弥散，同样无法识别注射部位^[9-11]^。③溶胶和琼脂注射定位法1996年Nomori等^[12]^利用不饱和胶原在体内可长时间驻留而没有并发症的特点，将之混合亚甲蓝，并加入一定量的碘苯六醇制备成溶胶，作为定位标记物在CT引导下注射到病变周围，这种物质可以在肺组织内驻留较长时间而不弥散，因为含有亚甲蓝在胸腔镜下能够窥及。Tsuchida等^[13]^将琼脂加热溶解注射到病变周围，琼脂凝固后变成坚硬的可以触知的结节，达到定位目的。但无论溶胶还是琼脂，最大的缺陷是制作复杂，且可导致残留。④碘油注射定位法1998年，Choi等^[14]^分别应用碘油、稀硫酸钡悬液和水溶性对比剂注射到病变或其周围，随即在X线透视辅助下实施胸腔镜手术，发现碘油的定位效果优于稀硫酸钡悬液和水溶性对比剂。虽然碘油在肺内短时间内不会发生明显弥散，这种方法同样建议在定位后即刻进行手术，且需在X线透视辅助下的手术限制了其推广。⑤放射性核素定位法2002年Burdine等^[15]^将放射性锝胶体硫核素在CT引导下注射到肺内病灶周围，在实施胸腔镜手术时，使用γ探头示踪放射性核素区域，继而楔形切除。此法虽然定位准确，但设备要求特殊，价格昂贵，且仅限于非常表浅的病灶。1993年，Shennib等^[16]^在术中应用超声波探测定位肺部微小病变，这是最早进行术中定位的报道，是一种无创、简易、经济的方法。然而这种方法依然有很大的局限性：①超声分辨率比较低，难以很好观察和定位 < 1 cm的病变尤其是纯磨玻璃样病变；②此操作受肺组织含气量的影响极大，不适用于部分哮喘和阻塞性肺病患者。此外，术中超声需要操作者具有较丰富的病变识别经验，由于这些缺点，此方法正逐渐被淘汰。

我们试行的术前利用放疗计划模拟CT穿刺联合亚甲蓝注射定位的方法，优势体现在：①术前定位过程中患者只进行一次CT扫描，射线辐射量小；②模拟定位后患者勿需马上进入手术室，可从容进行术前准备；③穿刺注射亚甲蓝在麻醉后进行，克服了既往所有术前定位方法对患者造成的心理恐惧；④因穿刺注射亚甲蓝后紧接着VATS手术，即便出现穿刺部位出血、气胸等情况，也可及时而轻易地处理；⑤注射亚甲蓝后的即刻手术，避免了亚甲蓝迅速弥散所导致的辨识困难。

然而，此方法也存在着一些局限性，包括：精确性稍低：相比较与CT引导下定位技术，此定位方法精确性稍低，因为CT引导下可以反复穿刺定位，能够确保金属钩或穿刺针到达病灶，所以精确性较高。此定位方法因为术前是模拟穿刺，模拟过程中每一个步骤之间都可能会产生一定的误差，原因有以下几点：①术中穿刺时体位与患者模拟穿刺时的体位难以保持完全一致，尤其是在背部进行穿刺时，由于模拟时是俯卧位，而穿刺时是健侧90度卧位，体位的改变难免会导致误差，甚至有个别病例直接导致定位失败，这是产生误差的主要原因。②由于穿刺时一般均采用水平穿刺或平行穿刺，当采用这两种角度进行穿刺时，病灶均恰好有肋骨遮挡，我们只能采取在相应肋骨上一肋间或下一肋间进行穿刺，导致穿刺针可能无法到达病灶本身，因而产生一定的误差。本组病例双侧上叶病灶的定位边距比较均衡，波动于4 mm-6 mm之间，给术者有益的定位指示，减少了病灶寻找时间。但两下叶病灶的定位平均边距于7 mm-10 mm间，距离明显增大。本研究中，共计4例病灶定位失败，病灶均位于下叶后部，术中见染色斑未能覆盖病灶。回顾该4例患者在模拟定位时取俯卧位且双手抱头，而麻醉后由于手术体位的要求，患者只能采取右侧90^0^卧位进行穿刺，因而模拟穿刺的皮肤标记点在体位变动后肯定产生移位。

鉴于上述定位方法的临床探索实践，作者有如下体会：①尽量使CT模拟体位与麻醉后穿刺体位保持一致；②定位上、中叶病灶，一般采取仰卧位，患者仰卧于手术床，手术者控制患者双手掌心向上，右手重叠于左手之下合拢，抱头于枕部；③定位下叶病灶时，我们总结上述3例定位失败的经验，将CT模拟时的患者体位改为手臂紧贴腋区自然下垂，与麻醉后手术体位一致，以尽量减少因手臂移动导致的胸部皮肤定位穿刺点的位置变化；④穿刺前请麻醉师配合控制双肺通气，使患者保持深吸气屏气状态，保持肺的膨胀状态与CT模拟时一致。⑤穿刺进针采取容易控制的角度，一般尽量平行或垂直于水平面；⑥注射亚甲蓝时应严格依据CT模拟时的数据，注意深度，在注射过程中边退针边注射，距脏层胸膜10 mm时再加注入0.2 mL为宜。此外，当穿刺点有肋骨或肩胛骨遮挡时，可以穿刺到最近距离，术中可在染色范围附近查找病灶，也可减少术中查找时间，避免因触摸肺组织时间过长导致局部肺水肿，加大查找病灶的难度。离心脏、大血管、膈肌等较近的病灶，由于穿刺风险极高，不宜应用此定位方法。

术前应用CT模拟机对肺部周围型微小病灶进行模拟穿刺定位，麻醉后依照模拟数据向病灶注射亚甲兰的定位方法准确率较高，可减少患者术前射线辐照，避免患者清醒穿刺的恐惧和相关并发症，麻醉后穿刺基本无并发症影响之忧，术中寻找病灶迅速。此方法对于肺部周围型微小病灶的定位，尤其是对清醒状态下穿刺惧怕及年老体弱的患者具有较高的临床价值，但仍然需要扩大研究样本，不断总结经验，逐步减少定位误差，并摸索特殊部位病灶的定位技术以完善方法。
